# Dysregulation of Pain- and Emotion-Related Networks in Trigeminal Neuralgia

**DOI:** 10.3389/fnhum.2018.00107

**Published:** 2018-03-20

**Authors:** Yanyang Zhang, Zhiqi Mao, Longsheng Pan, Zhipei Ling, Xinyun Liu, Jun Zhang, Xinguang Yu

**Affiliations:** ^1^Department of Neurosurgery, Chinese PLA General Hospital, Beijing, China; ^2^Department of Radiology, Chinese PLA General Hospital, Beijing, China

**Keywords:** trigeminal neuralgia, voxel-based morphometry, resting state functional connectivity, magnetic resonance imaging, chronic pain

## Abstract

Classical trigeminal neuralgia (TN) is a severe neuropathic facial pain disorder associated with increased risks of anxiety and depression. Converging evidence suggests that chronic pain pathophysiology involves dysfunctional pain-related and emotion-related networks. However, whether these systems are also among the culprit networks for TN remains unclear. Here, we aimed to assess TN-related anatomical and functional brain anomalies in pain-related and emotion-related networks. We investigated differences in gray matter (GM) volume and the related resting-state functional connectivity (rsFC) between 29 classical TN patients and 34 matched healthy controls. Relationships between brain measurement alterations, clinical pain and emotional states were identified. A longitudinal observation was further conducted to determine whether alterations in the brain could renormalize following pain relief. Reduced GM volumes in the bilateral amygdala, periaqueductal gray (PAG) and right insula were found in TN patients compared with healthy control subjects. Whole-brain rsFC analyses with the four above-mentioned anatomical regions as seeds identified three significantly altered functional circuits, including amygdala-DLPFC, amygdala-mPFC and amygdala-thalamus/putamen circuitry. The amygdala-DLPFC and amygdala-mPFC circuits were associated with clinical pain duration and emotional state ratings, respectively. Further longitudinal analysis found that rsFC strength abnormalities in two fronto-limbic circuits (left amygdala/left DLPFC and right amygdala/right PFC) were resolved after pain relief. Together, structural and functional deficits in pain-related and emotion-related networks were associated with TN patients, as demonstrated by our multimodal results. Pain relief had protective effects on brain functional connectivity within fronto-limbic circuits. Our study provides novel insights into the pathophysiology of TN, which may ultimately facilitate advances in TN intervention.

## Introduction

Trigeminal neuralgia (TN), a severe neuropathic pain disorder, is estimated to affect one in 15,000–20,000 people worldwide (Katusic et al., 1991; Mueller et al., 2011), and with an even higher prevalence of TN in expanding demographics, including aging individuals (Wang et al., 2015). TN is characterized by highly intense electric shock-like pain in one or more trigeminal distributions (Truini et al., 2005; DeSouza et al., 2015). The paroxysmal pain may either occur spontaneously or be provoked by innocuous sensory stimuli and movements, with no pain between paroxysms. As the disorder progresses, pain may become frequent and sustained, increasing the risk of anxiety and depression and greatly diminishing quality of life (Meskal et al., 2014; Wu et al., 2015).

It has been documented that chronic pain including TN is maladaptive for the brain (Apkarian et al., 2009, 2011; Baliki et al., 2011). Indeed, in addition to the brain’s normal activity, the brain of TN patients is continuously processing the barrage of salient and painful input, disturbing the brain structurally and functionally. It has been assumed that the brain dysfunction is causally involved in the development of chronic pain and associated mental comorbidity (Ploner et al., 2017). In fact, chronic pain including TN patients is often associated with an increased tendency toward depression and anxiety (Baliki and Apkarian, 2015; Wu et al., 2015). Under this circumstance, a better understanding of the brain changes following chronic TN may provide novel insight into the pathophysiologic mechanisms underpinning TN, which in turn, may facilitate advances in intervention.

Accumulating evidence form animal and human studies has confirmed the clinical relevance of brain pain-modulatory and pain-integrative regions in chronic pain (Ossipov et al., 2014), leading to the postulation that the pain-related system may also play a role in TN. Consistently, several structural imaging studies have demonstrated that TN is involved in gray matter (GM) changes in the periaqueductal gray (PAG), a critical component of the pain-related system (Knight and Goadsby, 2001). Furthermore, recent evidence reinforces the idea that the emotion-related network, which is involved in emotion, behavior and learning functions, is important for chronic pain. As a hub for the emotion-related network, the amygdala is associated with emotional learning, anxiety and stress regulation and exhibits smaller volume and altered connectivity during the transition to chronic pain (Vachon-Presseau et al., 2016). Indeed, the incidence of clinical depression and anxiety in TN patients is estimated to be nearly three times that observed in matched controls (Wu et al., 2015). Such negative emotional valence in TN patients also implicates the involvement of emotion-related circuitry. Of further note, brain imaging-based studies from other chronic pain conditions have suggested that dysfunction of the pain-related and emotion-related networks might be the neural substrates of pain chronification (Denk et al., 2014; Tracey, 2016). However, for TN, empirical evidence of brain changes in the pain-related and emotion-related systems is sparse, and is mainly obtained from studies using a single brain imaging modality. Importantly, if TN is indeed play a role in changing the pain-related and emotion-related systems, studies are needed to elucidate whether these brain changes are at least partially reversible following pain relief. Thus, a comprehensive examination of the involvement of the pain-related and emotion-related systems in TN needs to be performed.

Using the meta-analytic tool Neurosynth (Yarkoni et al., 2011), previous studies identified pain-related network including the insula, thalamus, mid-brain (PAG), anterior cingulate cortex and somatosensory area (Hashmi et al., 2013). The emotion-related network mainly includes amygdala, hippocampus, orbitofrontal cortices and operculum and dorsal, ventral and rostral regions of the medial PFC (Hashmi et al., 2013). In this study, we used a multimodal neuroimaging approach to test our hypothesis that TN is associated with the structural and functional changes within the above-mentioned pain-related and emotion-related networks. Using voxel-based morphometry (VBM) analyses of high-resolution structural MRI, we identified differences in regions of GM. Subsequently, using these regional morphological differences as seeds, we performed resting-state functional connectivity (rsFC) analyses to elucidate aberrant functional circuits or networks related to TN. We further hypothesized that the alterations in brain measurements that we identified should be associated with clinical pain and emotional states. Moreover, in contrast with other neuropathic pain syndromes, TN can be readily relieved by the microvascular decompression (MVD) surgery. Thus, based on longitudinal observation, we also tested TN patients after successful treatment to determine whether changes in their brains could renormalize.

## Materials and Methods

### Subjects

This study included 63 subjects: 29 consecutive patients (19 women and 10 men; mean age ± SD: 48.1 ± 11.9 years) scheduled to undergo MVD surgical procedures for the treatment of classical TN and 34 control subjects (healthy controls, HCs) with similar distributions of age, gender and years of formal education (21 women and 13 men; mean age ± SD: 43.3 ± 10.1 years). All patients had right-sided pain and met the criteria of the International Headache Society for TN. No patients had undergone prior MVD surgery or other treatments (i.e., gamma knife radiosurgery) for TN or received tricyclic antidepressants, opioids, or serotonin/norepinephrine reuptake inhibitors. Individuals were excluded if they had a history of other chronic pain conditions, psychiatric disorders, stroke/cerebrovascular ischemia, any other neurological or sensory deficits or TN attributed to another disorder. Written informed consent was obtained from each participant prior to study inclusion, and the study was approved by the local ethics committee of the Chinese People’s Liberation Army (PLA) General Hospital.

All subjects were asked to draw the extent of their neuralgia on a visual analog scale (VAS, 0–10, where 0 = no pain and 10 = maximum imaginable pain). The 17-item Hamilton Depression Rating Scale (HAMD) and the 14-item Hamilton Anxiety Rating Scale (HAMA) were used to quantify the depression- and anxiety-related symptoms of the subjects, respectively. The HAMD was administered to each participant by a psychiatrist using the Structured Interview Guide for Hamilton-Depression interview format (Williams, 1988). The HAMA was administered by the same psychiatrist immediately after the HAMD interview. The TN medication statuses were also recorded for each patient.

Additionally, a follow-up subset (*n* = 10; 7 women and 3 men; mean age ± SD: 49.3 ± 9.8 years) of pain-relieved patients was subjected to pain ratings, emotional evaluations and MRI scans similar to the preoperative protocol approximately 4–6 months after MVD surgery.

### Image Acquisition

TN patients stopped their pain medication for at least 24 h prior to MRI scan. MRI data acquisition was performed on a GE750 3.0 T scanner with an eight-channel phase array head coil. High-resolution structural images were collected using a sagittal Fast Spoiled Gradient-Echo (FSPGR) sequence with the following parameters: repetition time (TR), 6.7 ms; echo time (TE), 2.9 ms; flip angle, 7°; slice thickness, 1 mm; no gap; 192 sagittal slices; field of view (FOV), 256 × 256 mm^2^ and voxel size = 1 × 1 × 1 mm^3^. The functional images were obtained using an echo-planar imaging (EPI) sequence with the following parameters: repetition time = 2000 ms, echo time = 30 ms, flip angle = 90°, thickness/gap = 3.5 mm/0.5 mm, slices = 36, field of view = 224 × 224 mm^2^, voxel size = 3.5 × 3.5 × 3.5 mm^3^, and a total of 240 volumes. During the scan, participants were fitted with soft earplugs and instructed to keep their eyes closed, to remain motionless, and not to think of anything in particular. After the scanning, a simple questionnaire indicated that no participants had fallen asleep.

### VBM Analysis

Structural data processing and analysis was performed with Statistical Parametric Mapping (SPM12^1^), including the VBM toolbox^2^ and Diffeomorphic Anatomical Registration Through Exponentiated Lie Algebra (DARTEL), using Matlab (The MathWorks, Natick, MA, USA). For preprocessing, images were bias-field corrected, segmented and registered to the standard Montreal Neurological Institute (MNI) space using the unified segmentation approach (Ashburner and Friston, 2005). Subsequently, GM segments were modulated by the nonlinear component of the transformation to allow for comparison of the absolute amount of tissue corrected for individual brain sizes (volume of GM; Good et al., 2001). Finally, the resulting images were smoothed with an isotropic Gaussian kernel of 6-mm full-width at half maximum (FWHM). To detect GM volume differences between TN patients and HCs, we performed nonparametric permutation tests based on 10,000 permutations using the Statistical nonParametric Mapping (SnPM) toolbox^3^ in SPM12, controlling for age, gender and education level. To control for multiple comparisons, we set the significance level of the cluster-forming threshold to 0.01 with a familywise error rate (FWE) corrected cluster of *P* < 0.05. Because of the small anatomical size of brainstem nuclei, we further conducted a region of interest (ROI) approach to restrict analyses to the brainstem using the Harvard-Oxford subcortical structural mask, a correction for multiple comparisons was performed within this mask separately.

### Functional Connectivity Analysis

The resting-state fMRI data were analyzed using Statistical Parametric Mapping (SPM12^1^) and Data Processing and Analysis for (Resting-State) Brain Imaging (DPABI; Yan et al., 2016). Similar to previous studies (Zhang et al., 2017a,b), the preprocessing included removal of the first 10 time points, slice timing and head motion correction, realignment, spatial normalization (to MNI space), spatial smoothing, nuisance covariates regression, line detrending and band-pass filtering (0.01–0.08 Hz).

Then, we performed functional connectivity analysis using the seed-based approach. Regions with significantly morphological differences between TN patients and HCs (see “Results” section) were used as seeds in the subsequent rsFC analysis. The rsFC map for each seed region of interest was obtained by computing whole-brain voxel-wise correlations associated with the mean time course of the seed. The correlation coefficient maps for each individual seed were further normalized with Fisher’s *r*-to-*z* transformation and spatially smoothed (FWHW = 6 mm). Group-level rsFC maps were obtained by performing one-sample *t*-tests on the z-maps for each individual seed. The significance level of one-sample *t*-tests was determined by the cluster-forming threshold of *P* voxel < 0.001 with an FWE corrected cluster of *P* < 0.05 using SnPM under SPM12.

Differences in rsFC z-maps between TN patients and HCs for each region of interest were separately examined using a general linear model (age, gender and education level as nuisance factors). Similar to anatomical analysis, multiple comparisons were also corrected using the nonparametric method in SPM12, we also set the significance level of the cluster-forming threshold to 0.01 with a FWE corrected cluster of *P* < 0.05.

### Brain-Behavior Relationships

To explore the relationships between altered brain imaging indices (GM volume and rsFC) and behavioral measures (pain intensity, pain duration, HAMA and HAMD scores), correlation analyses were performed using mean values of GM volume or rsFC strengths within regions showing significant group differences against behavioral measures, controlling for age, gender and education level.

### Brain Changes Following Pain Relief

To determine whether GM volume and rsFC strength abnormalities associated with TN are resolved after pain relief, paired sample *t*-tests were performed to compare pre- and post-treatment (4–6 months after MVD) brain imaging indices (GM volume and rsFC) using region of interest (ROI)-based VBM and rsFC analyses. Values of GM volumes and rsFC strengths for each follow-up participant were extracted from ROI masks derived from the above-identified clusters that showed significant structural and functional alterations in TN patients. We further investigated whether the reversibility in functional connectivity correlated with TN duration.

## Results

### Subject Demographics

Table 1 summarizes the clinical and demographic characteristics of the study participants. No significant differences in age, gender, years of education or head movement between TN patients and HCs were found. However, TN patients showed significantly elevated HAMA and HAMD scores.

**Table 1:** Demographics and clinical data of all subjects.

	TN	HC	Group difference
	(*n* = 29)	(*n* = 34)	*P* value
Age (years)	48.14 ± 11.89	43.32 ± 10.07	0.087
Male/female	10/19	13/21	0.758
Education (years)	11.21 ± 3.99	11.53 ± 4.20	0.757
Duration of pain (years)	6.02 ± 4.35	NA	NA
VAS score	6.31 ± 1.15	NA	NA
HAMD score	3.79 ± 1.76	0.29 ± 0.46	<0.001
HAMA score	3.55 ± 1.18	0.26 ± 0.45	<0.001
Head motion (FD)	0.122 ± 0.047	0.130 ± 0.043	0.498
GM volume	0.332 ± 0.034	0.345 ± 0.032	0.122
Medication (CBZ/CBZ&GBP)	24/5	NA	NA

*Abbreviations: HC, healthy control; TN, trigeminal neuralgia; VAS, visual analog scale; HAMA, Hamilton Anxiety Rating Scale; HAMD, Hamilton Depression Rating Scale; FD, frame displacement; GM, gray matter; CBZ, carbamazepine; GBP, gabapentin; NA, not applicable*.

### GM Volume Between TN Patients and HCs

**Figure 1:**
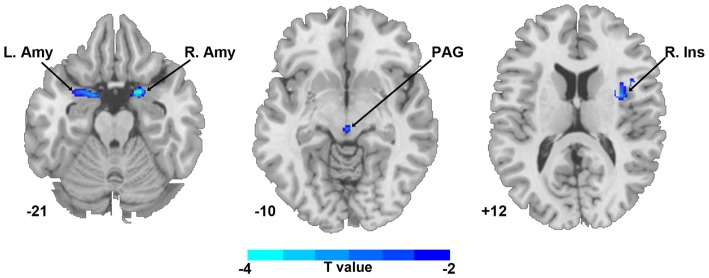
Regional gray matter (GM) volume differences in bilateral amygdala, PAG and right insula between TN patients and healthy controls (*P* < 0.05, corrected). The color bar displayed *t*-values. Abbreviations: Amy, amygdala; Ins, insula; L, left; PAG, periaqueductal gray; R, right; TN, trigeminal neuralgia.

Compared with HCs, the TN patient group showed smaller GM volumes in the bilateral amygdala, PAG and right insula (*P* < 0.05, FWE corrected; Figure 1 and Table 2); no significant difference in the mean whole GM volume between the groups was detected.

**Table 2:** Brain regions showing significant gray matter volume difference between trigeminal neuralgia (TN) patients and matched healthy controls.

	Cluster size	Peak MNI coordinate			Peak
Brain regions	(voxels)	*x*	*y*	*z*	*T*-value
Right insula	183	6	0	18	−3.46
Left amygdala	172	−19	3	−21	−4.04
Right amygdala	141	18	4.5	−22	−4.68
Periaqueductal gray	104	1.5	−24	−10	−3.75

### Resting-State Functional Connectivity Between TN Patients and HCs

**Figure 2:**
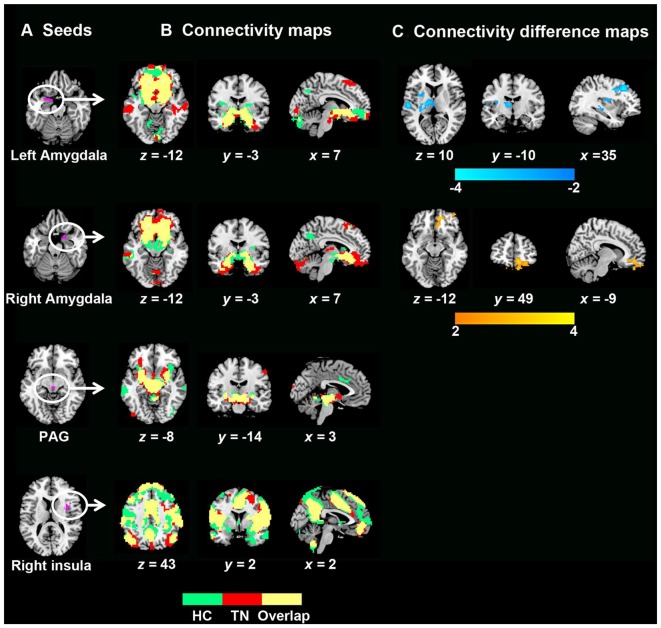
GM volume and related resting-state functional connectivity (rsFC) differences between TN patients and healthy controls. **(A)** Seed regions for rsFC analyses. **(B)** Functional connectivity patterns in TN patients and healthy control subjects. Green: healthy controls; red: TN patients; yellow: overlaps. **(C)** Functional connectivity differences between the two groups (*P* < 0.05, corrected). The color bar displays *t*-values. To note, between-group differences in rsFC analyses were only found related to seed of amygdala. Abbreviations: HC, healthy control; PAG, periaqueductal gray; TN, trigeminal neuralgia.

The seed-based FC maps of each group are presented in Figure 2. Visual examination indicated that both TN patients and HCs exhibited remarkably similar rsFC patterns despite some differences in strength. Further between-group comparisons revealed weaker connectivity strengths in TN patients relative to HCs between the left amygdala, left thalamus and putamen. A weaker connectivity between the left amygdala and left dorsolateral prefrontal cortex (DLPFC) was also observed. Additionally, TN patients exhibited enhanced connectivity between the right amygdala and right PFC (medial and orbital cortices; Figure 2 and Table 3). However, no significant rsFC differences were found between TN patients and HCs for the PAG or right insula seeds. Furthermore, rsFCs between the seed and each individual cluster were considered circuits, and TN was mainly associated with abnormalities in fronto-limbic circuits. Next we investigated the relationship between differences in functional connectivity and decreased GM volumes observed in the bilateral amygdala, and found no significant correlation between these two brain measurements (Supplementary Figure S1).

**Table 3:** Brain regions showing significantly different resting-state functional connectivity (rsFC) in TN patients.

		Cluster size	Peak MNI coordinate			Peak
Seed regions	Regions of difference	(voxels)	*x*	*y*	*z*	*T*-value
Left amygdala	Left thalamus/putamen/Superior temporal gyrus	540	54	−18	9	−4.06
	Left superior/middle frontal gyrus	238	−36	24	42	−4.20
Right amygdala	Medial frontal gyrus/orbital/rectal gyrus	297	36	54	0	4.03

### Brain-Behavior Relationships

**Figure 3:**
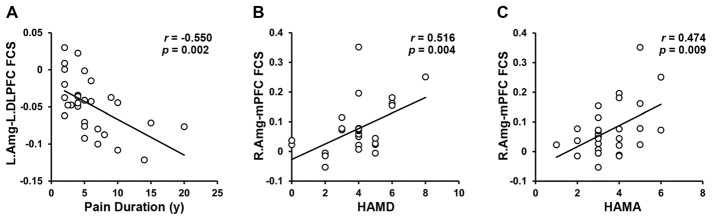
Brain–Behavior Relationships. The FCS of the left amygdala to the left DPFC was negatively correlated with the pain duration. **(A)** The FCS of the right amygdala to the mPFC was positively correlated with HAMD **(B)** and HAMA **(C)** in the TN patients. Abbreviations: L, left; Amg, amygdala; DLPFC, dorsolateral prefrontal cortex; FCS, functional connectivity strength; R, right; mPFC, medial prefrontal cortex; HAMD, Hamilton Depression Rating; HAMA, Hamilton Anxiety Rating.

A total of three amygdala-related circuits that exhibited altered rsFC strengths were identified from the four seeds of GM volumes differences between TN patients and HCs. The rsFC strengths of the left amygdala and left DLPFC were strongly negatively correlated with pain duration. Furthermore, both the HAMD and HAMA scores were positively correlated with rsFC strength between the right amygdala and right PFC (Figure 3). However, no relationship was observed between behavioral measures and GM volumes in regions showing between-group differences. Thus, these data indicate that deficits in fronto-limbic circuits are related to the clinical presentations of TN patients from both pain and emotional perspectives.

### Normalization of Fronto-Limbic Connectivity After Effective Pain Treatment

**Figure 4:**
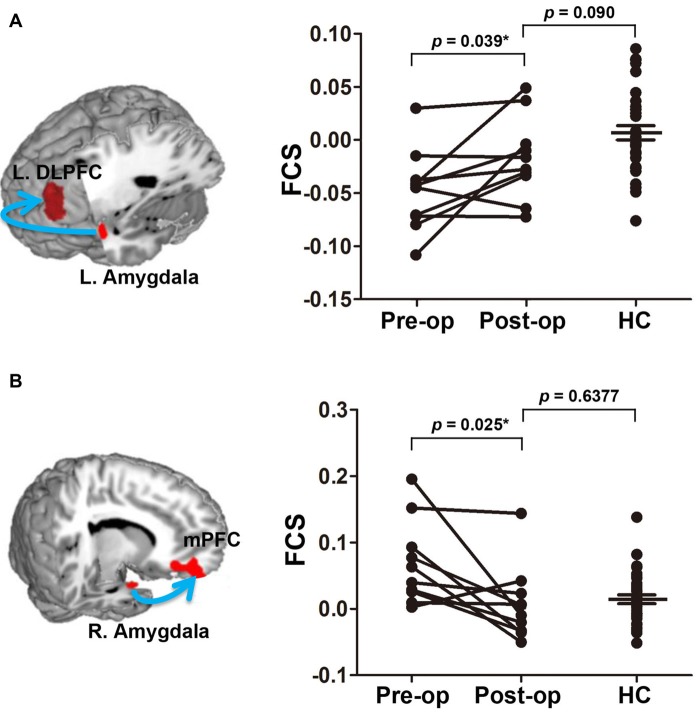
Normalization of the FCS of two fronto-limbic circuits after effective treatment. The left amygdala-DLPFC and right amygdala-mPFC circuit are illustrated in **(A)** and **(B)**, respectively. Arrow illustrates the rsFC between the seed and the target region but is not meant to suggest directionality. Data plotted are mean FCS values of the functional circuit pre- (pain) and post-operation 4–6 months (pain relief) in TN patients. After treatment, the FCS values are no longer different from healthy controls. Asterisks indicate that the *P* value is got from paired-sample *t* test. Abbreviations: L, left; DLPFC, dorsolateral prefrontal cortex; FCS, functional connectivity strength; R, right; mPFC, medial prefrontal cortex; pre-op, pre-operation; post-op, post-operation; HC, healthy control.

After effective treatment, the rsFC strength abnormalities in two fronto-limbic circuits (left amygdala/left DLPFC and right amygdala/right PFC) resolved such that the rsFC strengths were no longer significantly different from those of the HCs (Figure 4). No significant changes in regional GM volumes were observed after effective treatment compared with those recorded before treatment. Thus, pain relief has protective effects on brain functional connectivity within fronto-limbic circuits. Additionally, the reversibility in functional connectivity did not significantly correlate with TN duration (Supplementary Figure S2).

## Discussion

In this study, we initially identified structural markers of TN in pain-related and emotion-related regions of the human brain, including the PAG, right insula, and bilateral amygdala. Using these four regions as seeds in subsequent rsFC analyses, three amygdala-related functional circuits that differed between TN patients and HCs were identified and associated with clinical pain duration and emotional state ratings. Further longitudinal analysis of patients with pain relief following effective treatment revealed that brain abnormality normalization is restricted to functional fronto-limbic circuits. As the pain- and emotion-related regions are involved in TN, further attention to these regions as potential therapeutic targets and therapeutic strategy guides are warranted.

### Gray Matter Volume Reduction Reflects Chronic Pain in TN

Growing evidence supports the concept that chronic pain is associated with dysregulation in descending pain modulation (Lewis et al., 2012; Ossipov et al., 2014). Consistent with a previous report of altered GM volume in pain modulation regions, we found decreased PAG volume in TN patients. PAG, acting as a hub for the descending pain-modulatory network, receives inputs arising in multiple areas, including the hypothalamus, the amygdala and the rostral anterior cingulate cortex, and communicates with medullary nuclei that send descending projections to the spinal cord (Basbaum and Fields, 1978; Ossipov et al., 2010). Previous animal research has shown that the expression of nerve injury-induced pain may ultimately depend on descending pain modulation, suggesting that dysfunction of descending inhibition plays a role in the transition from acute to chronic pain (De Felice et al., 2011). Moreover, Knight and Goadsby (2001) found that electrical stimulation of the PAG could inhibit trigeminal nociceptive input and further proposed that PAG dysfunction might lead to the disinhibition of trigeminal afferents. Our findings of reduced GM volume in the PAG further confirmed that anatomic impairments in this region might underpin TN pathogenesis.

Importantly, we showed that the TN patients exhibit significant reductions in their GM volume in corticolimbic regions, including the insula and bilateral amygdala. Consistent with our study, previous structural imaging studies have shown corticolimbic volume decreases in TN patients. The amygdala plays important roles in the processing and regulation of emotion and is proposed to be a critical component of the pain matrix (Boakye et al., 2016). Given the connections between the amygdala and the pain-modulatory system, the amygdala may significantly contribute to the integration of pain and associated responses, such as anxiety and fear (Ossipov et al., 2010; Tracey, 2016). Thus, a reduced amygdala volume may conceivably impair its capacity to drive descending inhibition, thereby contributing to heightened pain experiences and negative emotional responses. Additionally, a longitudinal observational study from back pain patients showed that the gene-chronic pain relationship was fully mediated by the indirect effect of reduced amygdala volume (Tracey, 2016; Vachon-Presseau et al., 2016). Genetically labeling the role of the amygdala in the maintenance and development of TN is an attractive concept, and further work is warranted to validate these possibilities. Of note, in line with studies on a variety of pain conditions (Gustin et al., 2011; Henderson et al., 2013; Krause et al., 2016), we found decreased GM in the insula cortex. Studies using functional imaging and intraoperative electrical stimulation have identified the role of this region in pain processing (Brooks et al., 2005; Kong et al., 2006; Peltz et al., 2011). Moreover, a previous study found aberrant insula activity in chronic pain patients and demonstrated its relationship with altered autonomic nervous system function (Malinen et al., 2010). Recently, Wang et al. (2018) also emphasized the role of insular abnormalities in the pathophysiology of TN. However, in contrast to our findings of structural abnormalities in right insula in TN patients, they found changes of cortical gyrification and associated rsFC in left insula. The differences of lateralization may be due to the different patient characteristics and the different analysis methods. For instance, the results reported by Wang et al. (2018) derive from a mix of both left-sided and right-sided pain patient groups. Additionally, assessing cortical gyrification instead of VBM may have contributed to the differences in results. Further studies with larger sample sizes and standardized analysis protocols would help clarify this discrepancy.

In general, our results are in good accordance with previous VBM studies on other chronic pain conditions, which mostly found decreased GM volume or density in pain-modulatory and corticolimbic regions (Obermann et al., 2009; Ruscheweyh et al., 2011; Krause et al., 2016). Such brain changes have repeatedly been shown to be partially reversible following pain relief (Rodriguez-Raecke et al., 2009). However, using an ROI approach, we found that no GM regions were renormalized following effective treatment, raising the possibility that abnormal GM volume in the pain- and emotion-related regions might be preexistent, thus predisposing individuals to the development of TN after peripheral trigeminal nerve injury (e.g., neurovascular compression in the trigeminal root). Alternatively, these seemingly permanent GM changes might be driven by the transient but repetitive, peripheral pain inputs, such as those documented in animal models of chronic pain (Kuner, 2010). Elucidation of the specific patterns of structural plasticity will require additional studies.

### Functional Reorganization Confined to Amygdala-Related Circuitry

We found significantly altered functional connectivity in amygdala-related emotional circuitry but not in circuitries related to the insula and PAG, which are primary regions associated with pain perception and modulation. Previous studies have consistently demonstrated that the representation of brain activity gradually shifts from nociceptive to emotion-related circuitry during pain chronification (Apkarian, 2008). The dissociation might mirror sensory pain properties less and instead mirror the enhancement of the complex emotional relevance of the condition (Apkarian, 2008; Apkarian et al., 2009; Hashmi et al., 2013). Indeed, we observed that alterations in the functional connectivity strength of prefrontal-amygdala circuitry were significantly correlated with increased depression and anxiety. Recently, a study of TN patients also did not find the changes of functional connectivity in PAG- and insula- related circuits (Tsai et al., 2018). To note, the pain modulatory pathways involve projections from PAG to brainstem nuclei, including the rostroventral medulla (RVM) and the locus coeruleus, to the dorsal horn of the spinal cord (Basbaum and Fields, 1978; Ossipov et al., 2010). A wealth of brain and spinal cord imaging studies has emphasized the roles of these pain modulatory pathways for chronic pain conditions (Denk et al., 2014). Whether TN is also related to the abnormalities of pain modulatory pathways needs further brainstem- and spinal cord- imaging studies.

Recent studies have emphasized that context and prior events are critical for shaping an emotional experience (Lindquist et al., 2012; Hashmi et al., 2013). Within this framework, the amygdala is associated with orienting motivational preferences to salient stimuli (Moriguchi et al., 2011), while the mPFC is involved in assigning meaning to sensory cues based on prior learning (Bar, 2009; Mitchell, 2009) and connecting episodic memory to the affective appraisal of sensory events (Roy et al., 2012). This result, along with our finding of enhanced functional connectivity of mPFC-amygdala circuitry, is in line with the theoretical framework proposed by Hashmi et al. (2013) emphasizing that complex emotional state perceptions in chronic pain patients are constructed from learning and the resultant memory traces of pain persistence.

Another major finding of this study was the reduced functional connectivity of DLPFC-amygdala circuitry in TN patients. The DLPFC is associated with the experience, localization and modulation of pain (Coghill et al., 1999; Lorenz et al., 2003) and may modulate pain perception through a “top-down” mechanism by reshaping cortical-subcortical pathways (Lorenz et al., 2003). Thus, disrupted DLPFC-amygdala circuitry might implicate a lack of inhibitory control of nociceptive input among TN patients. Specifically, such disruption was dependent on pain duration, suggesting that chronic pain itself increasingly alters brain pain-control circuitry. Fortunately, this altered functional circuitry could be renormalized following effective treatment. It is worth noting that the reversibility in functional connectivity did not significantly correlate with TN duration. One possible reason may be that the small sample size has limited power to detect significant correlations. It is also possible that the correlations between reversibility in functional connectivity and TN duration may be biologically complicated rather than linear.

We also observed altered functional connectivity of amygdala-thalamus/putamen circuitry in TN patients. The thalamus is a pain region involved in affective and sensory processes related to pain (Bushnell and Duncan, 1989; Tracey, 2005), and thalamic atrophy and abnormal chemistry within this region are commonly associated with chronic pain (Apkarian et al., 2004, 2005). The putamen, a major site of cortical and subcortical inputs into basal ganglia, is frequently activated during pain and is associated with pain-related motor response processing (Coghill et al., 1994; Starr et al., 2011). For TN, several structural imaging studies have demonstrated anatomic changes in the thalamus and putamen (Gustin et al., 2011; DeSouza et al., 2013). Given that TN patients often restrict facial movements, such as chewing, to avoid pain attack triggers (Bennetto et al., 2007), reduced functional connectivity in amygdala-thalamus/putamen circuitry may partially reflect abnormal motor behaviors in TN. Moreover, such abnormality could not be reversed, which is consistent with clinical observations that patients still limited their facial movements despite pain relief following treatment.

To note, the weaker rsFC of left DLPFC-amygdala and enhanced rsFC of right mPFC-amygdala circuitry mirror that the right-sided TN affects brain in a lateralized manner. We propose that such phenomena results from: (1) the organization of the pain-related pathways predominately target the contralateral hemisphere; and (2) from the functional lateralization of amygdala potentially involving differential rsFC patterns in left- and right-sided hemisphere (Baas et al., 2004). Consistently, animal studies have showed that the left/right pain impacts differentially on cognitive behavior, and such observation is associated with the asymmetrical functions of forebrain structures (Leite-Almeida et al., 2012, 2014). In our study, including only right-sided TN patients, we could not investigate the lateralized effect of pain on the brain. Moreover, we did not examine the structural and functional asymmetries within bilateral hemispheres related to TN. To draw a more generalized conclusion, the lateralization of pain effects on the brain warrants further studies including both right- and left-sided pain populations.

For the rsFC analysis, we utilized ROIs derived from VBM results as seed regions. Although the structural changes did not significantly correlated with changes in functional connectivity, findings supported by these two modalities, would take advantage of the cross-information from each modality, thereby potentially enhancing power to detect imaging signature for TN (Geng et al., 2017). As brain function is known to be highly dependent on underlying structural features (Honey et al., 2007), it is expected that GM volume loss will be coupled with altered functional connectivity. On the other hand, previous studies demonstrate that intracellular cascades as a function of chronic pain can manifest as facilitated excitatory transmission and depressed inhibition of the responses to noxious stimuli, leading to the GM or neurons changes (Woolf and Salter, 2000). Such changes in intracellular communication may affect the functional connectivity between the regions of GM volume loss and other brain regions.

### Limitations and Future Directions

First, because the subjects were relatively heterogeneous and the sample size in each cohort was small, the detection power was considerably reduced. Indeed, in the analysis of detecting GM and functional differences between TN patients and HCs, no clusters survived at the voxel-wise thresholds of 0.001. Thus, we used the voxel-level correction threshold of 0.01 to balance the control over false positives and the maintenance of sufficient power to detect differences. This exploratory investigation of GM and functional changes needs to be replicated in larger cohorts. Second, functional brain networks constructed from the rsfMRI data were largely constrained by structural white matter pathways (Honey et al., 2009). Thus, further studies combining diffusion tensor imaging could facilitate uncovering the structure-function relationships in TN patients. Finally, although TN patients stopped carbamazepine for at least 24 h prior to MRI scan, the influence of medication on brain function cannot be completely ruled out. Studies combining a much larger cohort stratified for drug subcategories are required to clarify this issue.

## Conclusion

By combining structural and functional imaging data from a cohort of TN patients and matched HCs, we identified GM volume differences in the pain- and emotion-related networks. Functionally, three altered fronto-limbic circuits were identified and associated with clinical pain duration and emotional state ratings. Further longitudinal analysis of brain alterations following pain relief resulted in the reversal of functional mPFC-amygdala and DLPFC-amygdala circuitry. Taken together, these multimodal results support the previously reported pain- and emotion-related networks deficits in TN patients. Perhaps more importantly from a therapeutic perspective, the directionality of TN-related effects observed at the brain level differed from those previously reported on peripheral trigeminal injury alone in TN patients.

## Author Contributions

YZ, ZM, LP, ZL, XL, JZ and XY contributed to the conception of the study and to acquisition, analysis and interpretation of data. All the authors drafted the work and revised it critically for important intellectual content. Final approval of the version to be published was performed by all authors.

## Conflict of Interest Statement

The authors declare that the research was conducted in the absence of any commercial or financial relationships that could be construed as a potential conflict of interest.

## Footnotes

^1^http://www.fil.ion.ucl.ac.uk/spm

^2^http://dbm.neuro.uni-jena.de/vbm/

^3^http://www.nitrc.org/projects/snpm/
