# Supplementary figures and images for: Dysregulation of Pain- and Emotion-Related Networks in Trigeminal Neuralgia
Source: Front Hum Neurosci. 2018 Mar 20;12:107. doi: 10.3389/fnhum.2018.00107 (PMC5890150; doi:10.3389/fnhum.2018.00107)

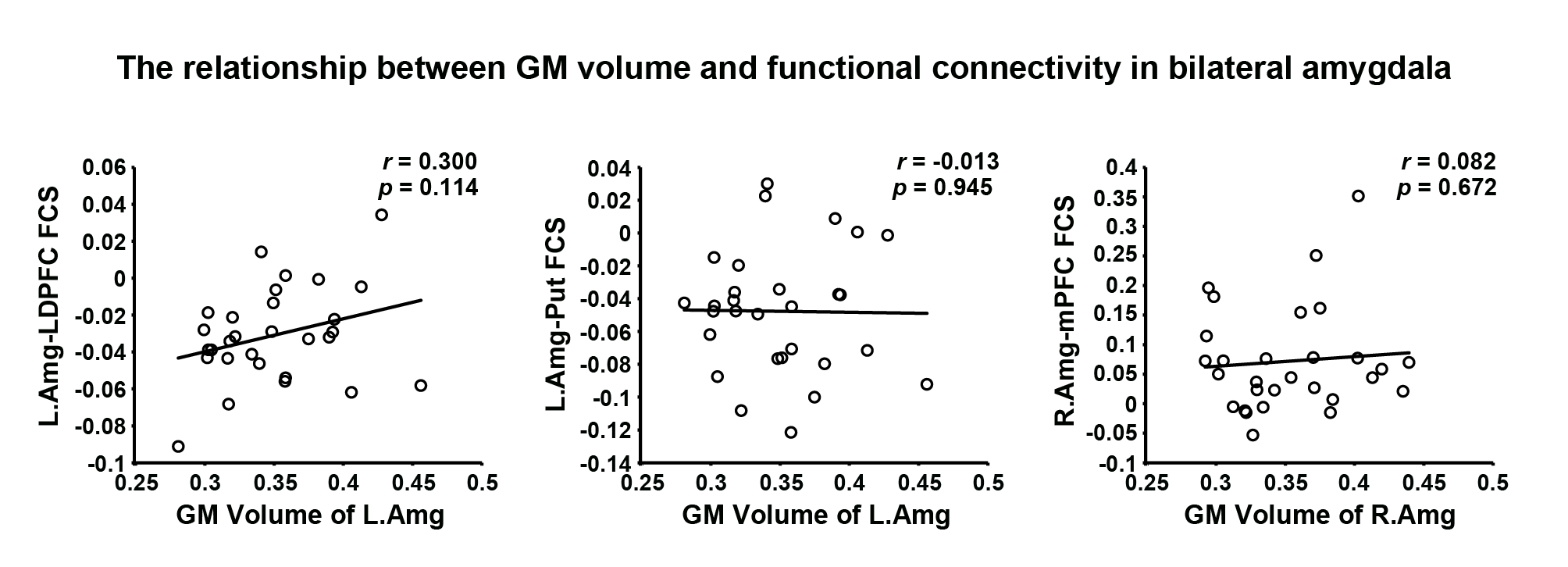

Supplement: Supplementary file 1 [file Image_1.TIF]

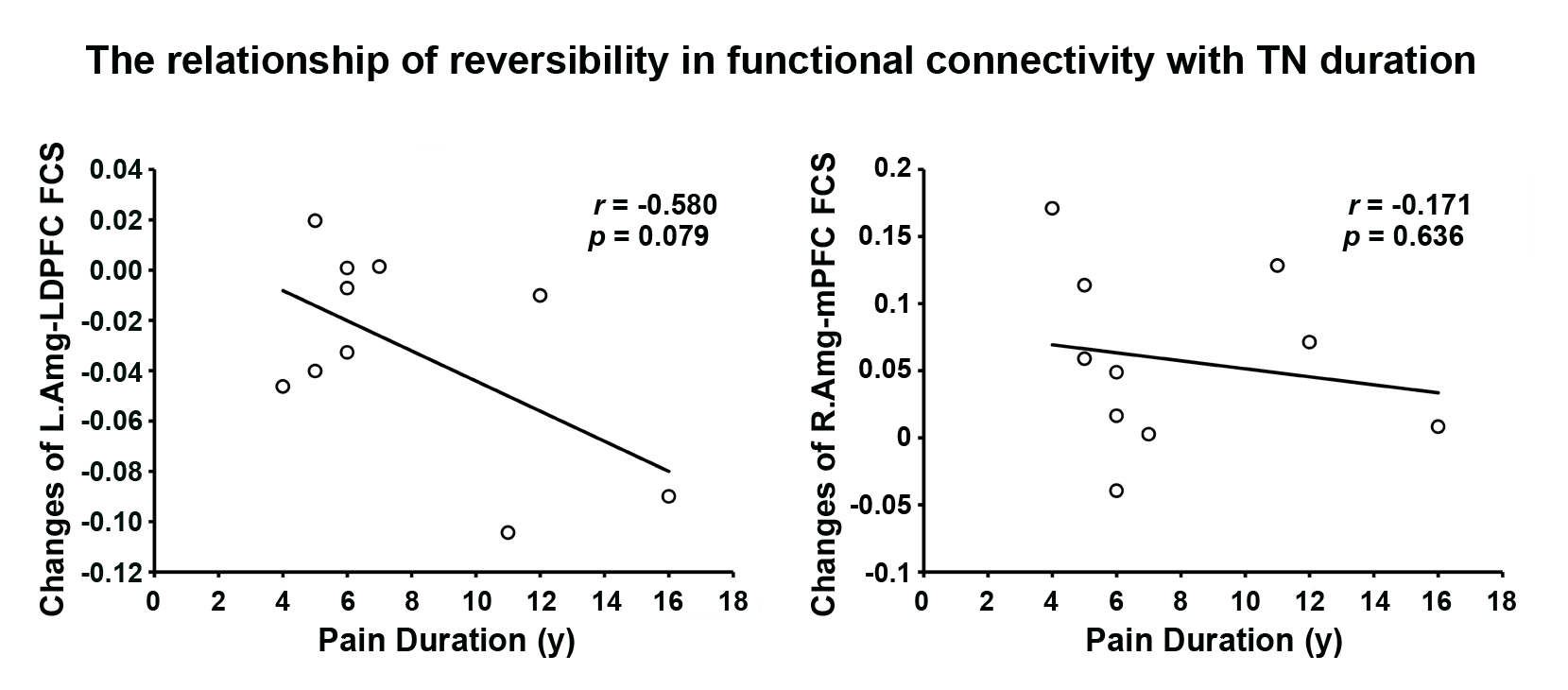

Supplement: Supplementary file 2 [file Image_2.TIF]
